# Checklist of British and Irish Hymenoptera - Trigonaloidea

**DOI:** 10.3897/BDJ.4.e7935

**Published:** 2016-04-15

**Authors:** Gavin R. Broad

**Affiliations:** ‡ The Natural History Museum, London, United Kingdom

**Keywords:** Britain, fauna, Trigonalyidae, Trigonalyoidea

## Abstract

**Background:**

The British and Irish checklist of Trigonaloidea comprises a single species, *Pseudogonalos hahnii*(Spinola), which is the only species in Europe.

**New information:**

Country-level distribution and nomenclature of *Pseudogonalos hahnii*are updated.

## Introduction

The Trigonaloidea is a small but cosmopolitan superfamily comprising approximately 110 species all classified in one family, the Trigonalidae. The single European species, *Pseudogonalos hahnii*(Spinola), is rarely collected in Britain and has not been found in Ireland. The superfamily is rather phylogenetically isolated (Klopfstein et al. 2013) and thus this is the smallest of the chapters of the Checklist of British and Irish Hymenoptera. The series of papers started with the checklists for Ceraphronoidea (Broad and Livermore 2014b), Evanioidea (Broad and Livermore 2014a) and the sawflies (Liston et al. 2014) and will be completed in 2016. 

The biology of trigonalids is unusual and is most similar to that of the ichneumonid subfamily Eucerotinae. The eggs are laid on foliage and only hatch when consumed by sawfly or lepidopteran larvae (in which respect they differ from eucerotines). Further development takes place as a hyperparasitoid of a primary parasitoid within the sawfly or lepidopteran larva. Sometimes development takes place as a parasitoid of a vespid larva if the secondary host is taken as prey by vespids. Some species have been reported to be primary parasitoids of sawfly larvae (biology summarised in Carmean and Kimsey 1998). *Pseudogonalos hahnii*has not been reared in Britain but there are European rearing records from several Lepidoptera species (summarised by Carmean and Kimsey 1998).

**Figure 1.**
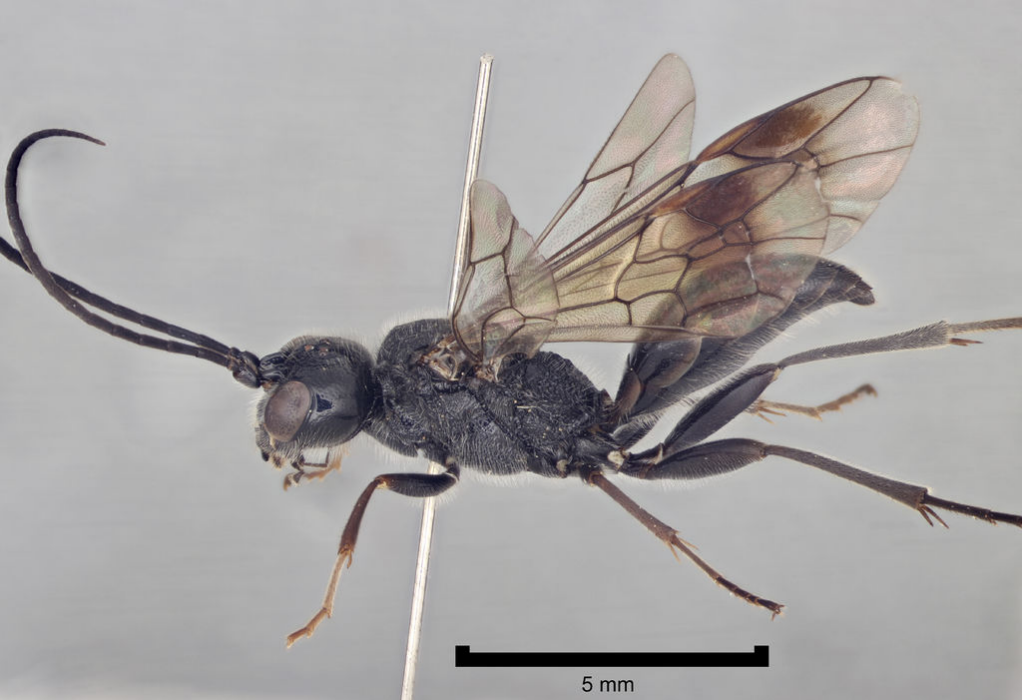
*Pseudogonalos hahnii*(Spinola), habitus view, England, Kent, Roundshill Park, 18.7.1998, coll. L. Clemons, NHMUK010209582.

*Pseudogonalos hahnii*is illustrated in Fig. 1. Trigonalids are sometimes misidentified as aculeate wasps, which they superficially resemble, but can be separated by the greater number of antennal segments (usually more than 18); characteristic wing venation; large, quadridentate mandibles (Fig. 2a); and the presence of plantar lobes on the tarsi (Fig. 2b); together with a suite of apomorphies, as detailed by Carmean and Kimsey (1998). 

## Materials and methods

See Broad (2014) for the background and general rationale behind the checklist. We have tried to account for every name on the 1978 checklist (Fitton et al. 1978) and all additions to the fauna since then have been referenced. The following conventions and abbreviations are used throughout (but not necessarily in this short chapter):

[***species***] taxon deleted from the British and Irish list

NHM Natural History Museum, London

# known introductions occurring only under artificial conditions

? status (including uncertain synonymy) or identification in the British Isles uncertain

misident. has been misidentified as this name

nom. dub. *nomen dubium*, a name of unknown or doubtful application

nom. ob. *nomen oblitum*, ‘forgotten name’, does not have priority over a younger name

nom. nov. *nomen novum*, a replacement name

nom. nud. *nomen nudum*, an unavailable name, with no type specimen

preocc. name preoccupied (junior homonym)

stat. rev. *status revocatus*, revived status (e.g., raised from synonymy)

unavailable not meeting the requirements of the International Code of Zoological Nomenclature

var. variety, only available as a valid name under certain provisions of the ICZN code

Photographs were taken using a Canon EOS 450D digital camera attached to a Leica MZ12 stereomicroscope and partially focused images were combined using Helicon Focus v.4.80 software.

## Checklists

### Trigonaloidea

#### Trigonalidae

Cresson, 1887

##### Notes

The alternative spelling of ‘Trigonalyoidea’ and ‘Trigonalyidae’, for the superfamily and family respectively, are often met with in the literature (e.g. Lelej 2003); according to Schnee (2011), Carmean and Kimsey (1998) and Aguiar et al. (2013), the correct spelling omits the ‘y’. Nomenclature from Carmean and Kimsey (1998), Lelej (2003) and Fauna Europaea (data compiled by M. Madl).

#### Pseudogonalos

Schulz, 1906

ABASTUS Guérin-Méneville, 1840 invalid
JEZOGONALOS Tsuneki, 1991
TRIGONALIS misident., misspelling

##### Notes

Currently classified in the subfamily Trigonalinae, but not assigned to a tribe within that subfamily (Carmean and Kimsey 1998).

#### Pseudogonalos hahnii

(Spinola, 1840)

Trigonalis hahnii Spinola, 1840
anglicana (Shuckard, 1841, *Trigonalys*)
europaea (Westwood, 1841, *Trigonalys*)
macquarti (Guérin-Méneville, 1842, *Abastus*)
nigra (Westwood, 1843, *Trigonalys*)
solitaria (Jacobs 1848, *Trigonalys*)
aterrima (Eversmann, 1849, *Trigonalis*)
phaeognatha (Enderlein, 1905, *Trigonalis*)
enslini (Torka, 1936, *Trigonalis*)
prudnicensis (Torka 1936, *Trigonalys*)

##### Distribution

England, Scotland, Wales

##### Notes

Listed in Fauna Europaea as *Trigonalis hahnii*. *Pseudogonalos *is treated as a separate genus on sound phylogenetic ground by Carmean and Kimsey (1998) (it is not even considered to be in the same tribe as *Trigonalys*). *Trigonalis *is an incorrect spelling of *Trigonalys*. Although the former range was evidently extensive, this species may now be restricted in Britain to southern England and South Wales, with the most recent published records being by Shaw (1990) and Howe and Howe (2005).

## Supplementary Material

XML Treatment for Trigonalidae

XML Treatment for Pseudogonalos

XML Treatment for Pseudogonalos hahnii

## Figures and Tables

**Figure 2a.**
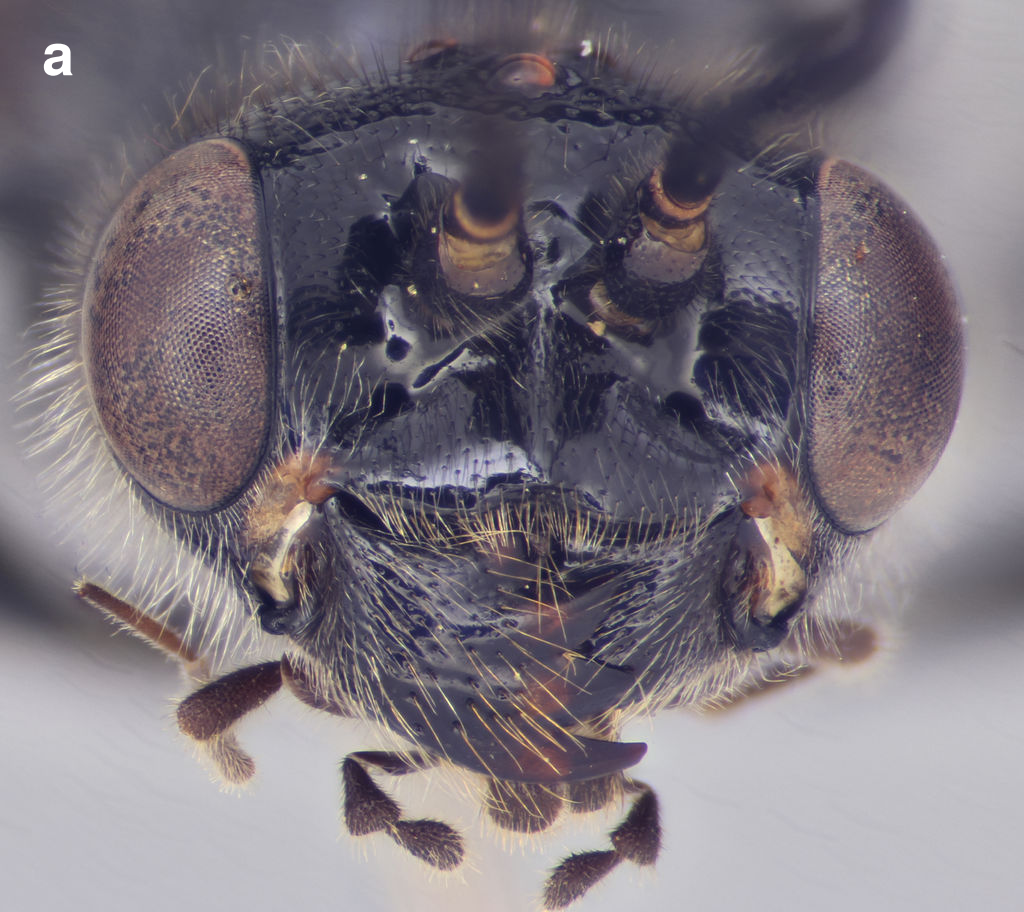
Face and mandibles

**Figure 2b.**
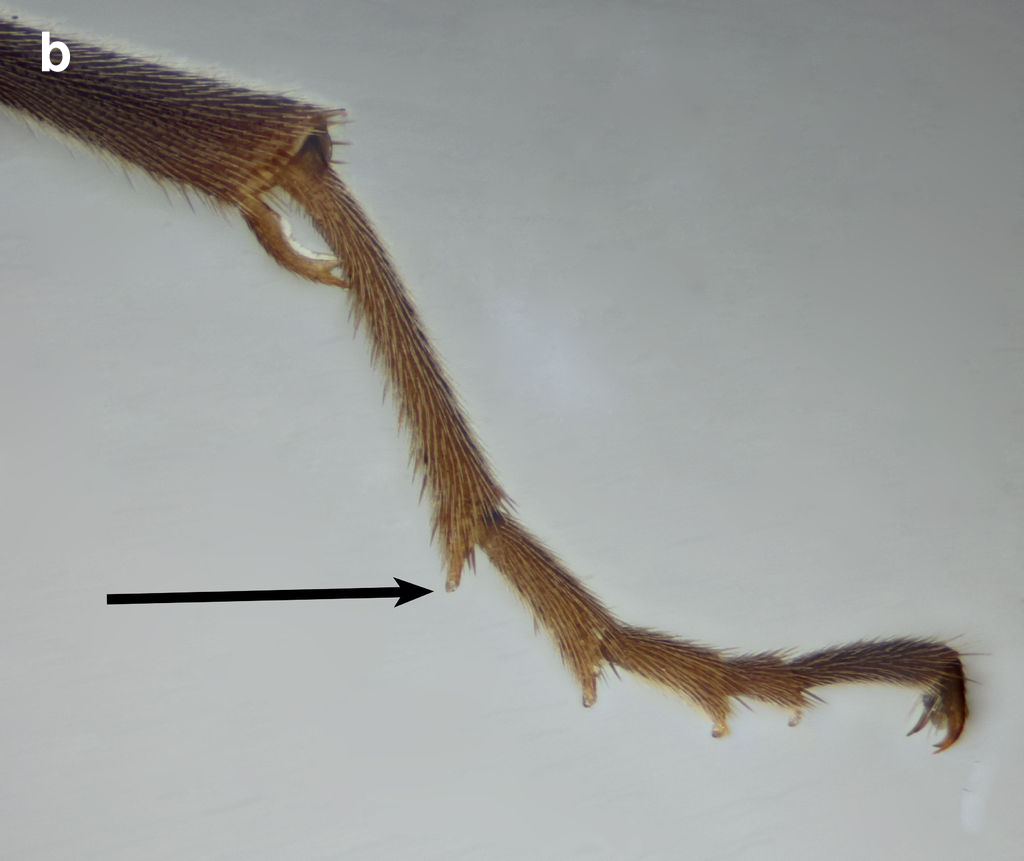
Fore tarsus with arrow pointing to a plantar lobe
